# Short Exposures to Phosphine Trigger Differential Gene Expression in Phosphine-Susceptible and -Resistant Strains of *Tribolium castaneum*

**DOI:** 10.3390/genes16030324

**Published:** 2025-03-10

**Authors:** Christos G. Athanassiou, Daniel Brabec, Morgan Olmstead, Nickolas G. Kavallieratos, Brenda Oppert

**Affiliations:** 1 Laboratory of Entomology and Agricultural Zoology, Department of Agriculture, Crop Production and Rural Environment, University of Thessaly, Phytokou Str., 38446 Nea Ionia, Greece; athanassiou@uth.gr; 2 USDA Agricultural Research Service, Center for Grain and Animal Health Research, 1515 College Ave., Manhattan, KS 66502, USA; daniel.brabec@usda.gov; 3 Department of Entomology and Plant Pathology, North Carolina State University, Raleigh, NC 27695, USA; molmste@ncsu.edu; 4 Laboratory of Agricultural Zoology and Entomology, Department of Crop Science, Agricultural University of Athens, 75 Iera Odos Str., 11855 Athens, Greece; nick_kaval@aua.gr

**Keywords:** CYP450 9e2, gene expression analysis, insect genetics, phosphine resistance, red flour beetle, RNA-Seq, stored-product insects, *Tribolium castaneum*

## Abstract

Background/Objectives: Phosphine resistance in insects involves a complex interplay of genetic and physiological factors, which are often poorly understood. Resistance to high concentrations of phosphine worldwide poses a formidable challenge for stored-product pest management and affects global food security. Understanding the genetic basis of phosphine resistance in the red flour beetle, *Tribolium castaneum*, is urgent because of the species’ status as a notorious insect pest of stored grains and their resistance to major classes of insecticides. In this study, we take advantage of *T. castaneum* as a model species for biological and genetic studies. Methods: To tease apart genetic mutations and the differential expression of genes responding to phosphine intoxication, we set up 16 different exposure tests to compare the effects of phosphine dose, exposure time, and sampling time on gene expression in phosphine-susceptible and -resistant *T. castaneum* adults. Results: We examined the enrichment of gene ontology terms in genes that were differentially expressed and found that the data further distinguished differences in gene expression by insect strain, phosphine dose, exposure time, and recovery from phosphine exposure. The gene-encoding cytochrome P450 9e2 was expressed more in phosphine-resistant compared to phosphine-susceptible insects under all treatment conditions and was significantly higher in expression in resistant insects that were sampled after short or long phosphine exposures. Therefore, this gene may serve as a new phosphine resistance marker in *T. castaneum* and can further be utilized as a diagnostic tool for resistance detection. Conclusions: These data are important to understand the complex molecular changes in insects that have reduced sensitivity to phosphine to develop new monitoring and resistance prevention strategies.

## 1. Introduction

Phosphine resistance in stored-product insects involves the complex interplay of genetic, metabolic, and physiological factors. Understanding these mechanisms is crucial for developing effective management strategies to mitigate phosphine resistance and ensure the sustainable control of insect pests in stored products. Given that phosphine is by far the dominant insecticide currently in use in stored-product protection globally, and considering its importance for international trade, any issue that deals with its efficacy is related to global food security [1]. Resistance to phosphine has been recorded in many parts of the world in a wide range of stored-product species via a series of surveys about different facilities and commodities [1,2].

High-level resistance to phosphine, which is generally known as “strong resistance”, has been described in several insect species worldwide and poses a significant challenge for stored-product pest management [3]. So far, strongly resistant populations have been recorded in different parts of the world in different beetle species, such as the rusty grain beetle, *Cryptolestes ferrugineus* (Stephens) (Coleoptera: Laemophloeidae), the granary weevil, *Sitophilus granarius* (L.) (Coleoptera: Curculionidae), the rice weevil, *Sitophilus oryzae* (L.) (Coleoptera: Curculionidae), the lesser grain borer, *Rhyzopertha dominica* (F.) (Coleoptera: Bostrychidae), the red flour beetle, *Tribolium castaneum* (Herbst) (Coleoptera: Tenebrionidae), and the khapra beetle, *Trogoderma granarium* Everts (Coleoptera: Dermestidae) [1,2,4,5]. The resistance mechanisms identified in various species, including Coleoptera and nematodes, have raised concerns about the long-term effectiveness of phosphine as a fumigant and the potential for cross-resistance to other pest control treatments [1,6,7]. Genetic factors supporting phosphine resistance can sometimes overlap with those governing resistance to other chemicals, leading to cross-resistance potential, such as the phosphine and deltamethrin cross-resistance seen in a strain of *T. castaneum* [8]. Thus, researchers have sought other control methods to mitigate phosphine resistance, such as another fumigant, sulfuryl fluoride, which has not demonstrated cross-resistance to phosphine in *R. dominica* [9].

The need to understand the genetic basis of phosphine resistance in *T. castaneum*, which is often utilized as a model species for genetic studies [10], is underscored by the species’ status as a notorious insect pest of stored grains and its development of resistance to major classes of insecticides [11]. Furthermore, the identification of these genetic loci provides valuable insights for resistance monitoring and management strategies in pest control [5,12,13]. Earlier studies indicated that the genetic basis of phosphine resistance in *T. castaneum* involves the *rph1* locus, associated with weak resistance, and the *rph2* locus, which acts synergistically with *rph1* to confer strong resistance [12,13,14]. Specifically, the *rph1* locus in *T. castaneum* is linked to the S349G mutation in the gene *cytochrome b5 reductase* (*cyt-b5-r*), while the *rph2* locus is linked to the P45S and P49S mutation in the *dihydrolipoamide dehydrogenase* (*dld*) gene [8,13,15]. Additionally, many genes related to mitochondrial functions are differentially expressed in phosphine-resistant and -susceptible *T. castaneum* [8].

Another prevailing mechanism of phosphine resistance in insects involves the reduced uptake of the fumigant, a process designated as active exclusion [16]. This resistance mechanism has been observed in various insect species, including stored-product pests, and has important implications for the long-term efficacy of phosphine as a fumigant for pest control at the post-harvest stages of durable agricultural commodity production [1]. Nevertheless, the links between this resistance mechanism and the genetic mutations indicated above are poorly understood.

Narcosis in insects refers to a state of reversible stupor or immobilization induced by various agents, including high doses of phosphine [17,18,19]. Narcosis may serve as a protective mechanism against the toxic effects of the gas [20] and can lead to a walking response in insects, further highlighting the physiological effects of narcosis [18]. The narcosis response in phosphine-resistant *T. castaneum* was incorporated into the development of a rapid diagnostic test for the evaluation of insect tolerance/resistance to phosphine [21] and is now commercially available for more than ten stored-product beetle species [22]. Nevertheless, a limitation of this diagnostic kit is that it requires live insects and the small-scale generation of phosphine on site for direct exposure [21,22,23], which may not be feasible in “real-world” fumigation. To further correlate insect immobilization to phosphine resistance, Sakka et al. [5] evaluated a large number of *T. castaneum* strains to connect the speed of immobilization with the speed of recovery. In that work, the authors clearly demonstrated that quick knockdown results in high mortality, and thus, slow or no knockdown is likely to correlate with increased recovery and act as an indicator of resistance.

Although the immobilization response of insects to phosphine within a 90 min exposure timeframe can be a reliable indication of susceptibility, the data that are available for the molecular diagnostics of resistance to phosphine using gene expression studies are based on longer exposures, i.e., 20 h or more [8]. Some markers may be nondetectable at longer exposure intervals because of the massive changes in gene expression that occur over time in both phosphine-susceptible and -resistant insects exposed to phosphine. Theoretically, shorter exposures will trigger short-term shifts in gene expression that can be differentiated based on phosphine susceptibility immediately following the exposure period. These genetic markers may be coopted into a rapid “COVID 19-type” diagnostic that can distinguish resistant from susceptible individuals. Similar tests have been developed so far for several species, such as the malaria mosquito, *Anopheles gambiae* Giles (Diptera: Culicidae) and the silverleaf whitefly, *Bemisia tabaci* (Gennadius) (Hemiptera: Aleyrodidae) [24]. Recently, Sakka et al. [25] successfully evaluated three TaqMan qPCR assay protocols for the rapid characterization of phosphine resistance in different strains of *T. castaneum* and *R. dominica*. Although this technology is a step towards the development of rapid diagnostic testing, the insects were tested without previous exposure to phosphine, indicating that this series of tests is a “grey area” of phosphine resistance characterization, and not an “on–off” classification protocol.

Hence, as a continuation of translating genetic research into diagnostic detection, in the current study we evaluated gene expression in adults from phosphine-susceptible and -resistant strains of *T. castaneum* exposed to low and high doses of phosphine for 15 and 90 min exposure times. In addition, gene expression was compared in individuals selected immediately post treatment or 120 min later. In this context, the current work is the first to assess the correlation between behavioral changes after short-term exposure to phosphine and molecular indicators to advance the creation of a rapid molecular diagnostic tool. The development of a rapid phosphine evaluation test will enable grain handlers and producers to identify potential phosphine-resistant insects in the field so that alternative control methods can be utilized prior to the selection of resistant populations.

## 2. Materials and Methods

### 2.1. Insects

The phosphine-susceptible and -resistant *T. castaneum* strains used in this study were the same as those used in a previous study [8,21]. At the time of the study by Oppert et al. [8], the LC_50_ of adults in the phosphine-susceptible strain was 1.35 ppm, whereas the phosphine-resistant strain had an LC_50_ of 309 ppm for phosphine. Adults < 1 mo old used in the test sand were reared in environmental chambers at 27.5 °C and 65% relative humidity (R.H.) on whole wheat flour with 5% brewer’s yeast [8,21,22].

### 2.2. Phosphine Treatments

We used a modified version of the Phosphine Tolerance Test (PTT) (Detia Degesch GmbH, Laudenbach, Germany) for phosphine exposure, based on a procedure from previous studies [22,23]. Briefly, 20 adults of each of the phosphine-susceptible (S) or -resistant (R) strains (separate sets of adults for each of the four biological replicates) were placed in a 100 mL plastic kit syringe and phosphine was injected at either 1000 (low, L) or 3000 (high, H) ppm. The insects inside the syringe were exposed for 15 or 90 min and were either frozen immediately after the specified treatment time, or after a recovery period of 120 min post-exposure time (PET, *). Five insects from each group were flash-frozen in liquid nitrogen and stored at −80 °C for sequencing. Notations used for treatments are summarized in Table 1.

### 2.3. Sequencing

To prepare for sequencing, *T. castaneum* adults from each treatment replicate group were weighed and averaged 8.54 mg of tissue per tube (i.e., five insects). Frozen adults were transferred into pre-loaded ZR Bashing Bead Lysis Tubes (Zymo Research) and ground with clean plastic pestles, after which lysis buffer was added and samples were macerated in a Bullet Blender 24 (Next Advance, Troy, NY, USA) at maximum speed for 3 min. Samples were extracted using a modified version of the Quick-RNA Tissue/Insect v1.06 kit (Zymo Research, Irvine, CA, USA), incorporating the optional DNase step, increasing centrifuge times, and adding an additional ethanol drying step. mRNA was extracted using a Poly(A) RNA selection v1.5 kit protocol (Lexogen, Vienna, Austria). Libraries were made using a CORALL RNA-Seq library Prep Kit, with the UDI protocol (Lexogen) modified to utilize the combinatorial 6nt i7 and i5 indices. PCR cycles were adjusted throughout the experiment to reconcile overamplification. Libraries were sequenced on two MiSeq V2 300 cycle runs, using 101 × 101 and 151 × 151 cycles, and a NextSeq 2000 P2 flowcell using 101 × 101 cycles (Illumina, Valencia, CA, USA). Paired reads and technical sequencing replicates were concatenated and merged using BBMerge (BBtools version 38.96, [26]). The total reads per treatment ranged from 8.3 to 15.2 million (Supplemental File S1). Raw reads were submitted to NCBI SRA accessions SRR25097208—SRR25097407.

### 2.4. Bioinformatic Analysis

For the bioinformatic analysis, we approached the analysis of the large and complex dataset by asking specific questions and including relevant sample replicates in separate analyses. Initially, we used an ANOVA analysis as described below to narrow the gene set to a specific statistical cutoff and then evaluated the gene ontology (GO) terms associated with the gene set to identify overall molecular functions that are specific to that dataset. We then focused on the expression patterns of specific genes to further understand the differences related to phosphine susceptibility, the dose or time of phosphine exposure, and recovery time. To achieve this, all sequence data were imported into Arraystar (DNASTAR, Madison, WI, USA) for differential gene expression analyses. Sequences were mapped to the *T. castaneum* genome (GCA_000002335.3) and were normalized via RPKM [27]. Selected treatment groups were analyzed for statistical significance using ANOVA (*p* < 0.01 or 0.05, as noted). GO overrepresentation tests were performed on each set of differentially expressed genes in ShinyGO ([28], FDR < 0.05), an online gene ontology enrichment tool. Results were limited to *T. castaneum* and the main GO categories: biological process (BP), cellular component (CC), and molecular function (MF).

## 3. Results

Insects in this study had been categorized previously in terms of their activity at 1000 or 3000 ppm exposure to phosphine over 15 to 90 min [21]. At 1000 ppm, the susceptible insects were immobile after 90 min, but the resistant insects were mostly active. At 3000 ppm, both the susceptible and resistant insects were mostly immobile, but the resistant insects recovered after 2 h, while the susceptible insects remained immobile. After seven days, the resistant insects were active, whereas the susceptible insects were not active (dead). In this study, we followed the immobilization study by sequencing data from the same insects after they were exposed for short (15 min) or long (90 min) exposure times at the previous low (1000 ppm) and high (3000 ppm) doses, and we also included those that were frozen immediately or 120 min PET. The comparison of gene expression in these treatment groups is discussed in the following sections.

### 3.1. Differential Gene Expression Among All Treatments in Both T. castaneum Strains

Gene expression was compared among all 16 treatment groups of phosphine-resistant and -susceptible *T. castaneum* adults exposed to low and high doses of phosphine for 15 and 90 min. We selected insects immediately post-phosphine exposure or 120 min later. There were 352 differentially expressed genes (significant at *p* < 0.01) among all treatment groups in the two insect strains, with the expression of 196 genes decreased (highlighted in blue) and 113 genes increased (highlighted in orange) in phosphine-resistant insects (Supplemental File S2, All_.01).

**Figure 1:**
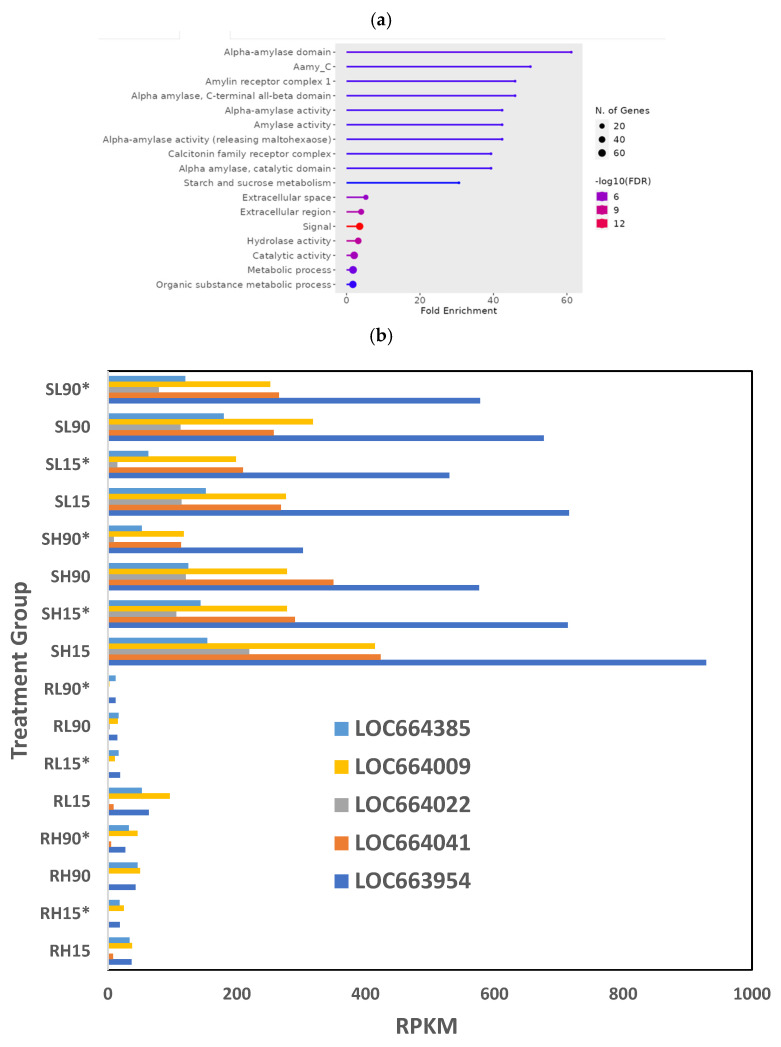

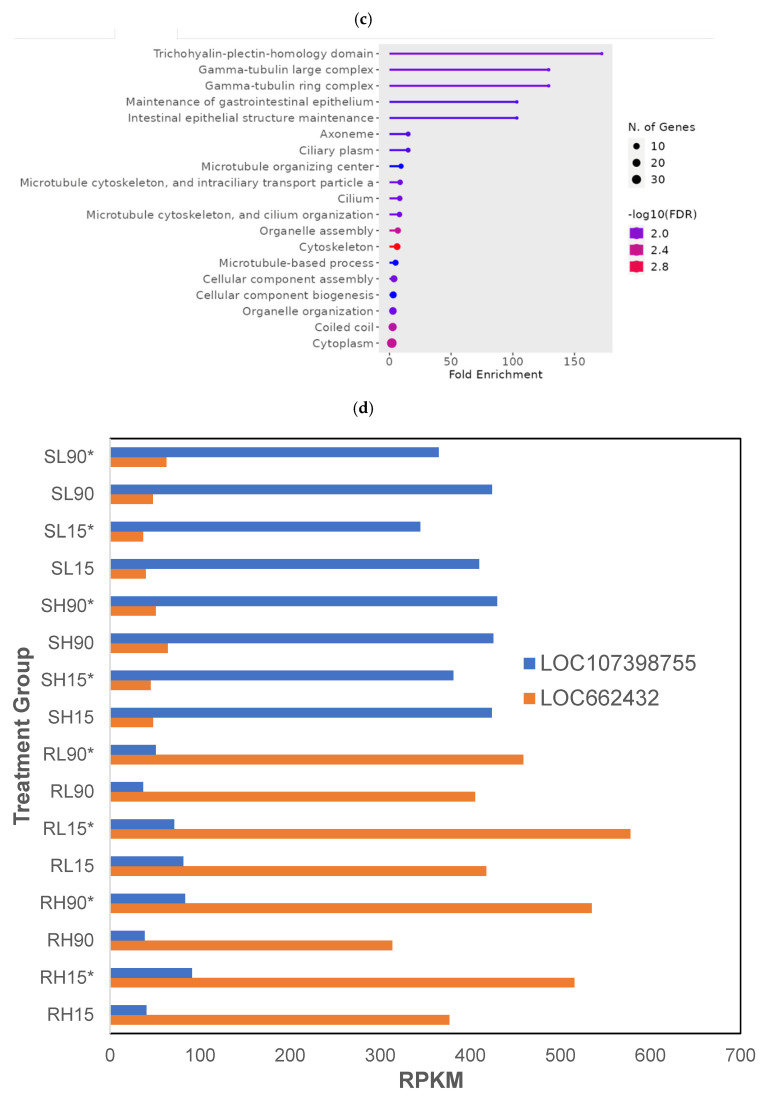
**Differences in gene expression among different treatments (dose, time, recovery) in phosphine-susceptible and -resistant *T. castaneum* adults.** Functional enrichment of GO terms as represented in ShinyGO plots from (**a**) downregulated or (**c**) upregulated genes. (**b**) Differential expression of α−amylase genes (LOC664385, LOC664009, LOC664022, LOC664041, LOC663954) in *T. castaneum* adults in all treatment groups. (**d**) Differential expression of *T. castaneum* genes LOC662432 (CYP9e2) and LOC107398755 (uncharacterized protein DDB_G0290685-like isoform X1) among all treatment groups. Groups with asterisks were sampled 120 min PET.

GO terms in the subset of downregulated genes essentially contained most of the downregulated digestive enzymes (Figure 1a), with glycosidases and hydrolases as the most enriched GO terms, “signal” as the most significant (lowest FDR) term, and “Catalytic activity” and “metabolic process” containing the most genes. Among the downregulated glycosidases, there were five a-amylase genes—LOC664385, LOC664009, LOC664022, LOC664041, LOC663954—expressed at lower levels in phosphine-resistant insects compared to phosphine-susceptible insects (Figure 1b). The downregulated genes in this dataset related to signaling were from the “calcitonin family receptor complex”, a group of receptors containing G protein-coupled receptors (GPCRs) and receptor-activity-modifying proteins (RAMPs; [29]).

Among the upregulated genes in phosphine-resistant *T. castaneum* adults, enriched GO terms came from genes involved in cellular restructuring (Figure 1c). The most enriched GO term in this group was the “trichohyalin-plectin-homology domain”, the GO term with the most genes was cytoplasm, and the GO term with the most significant FDR was cytoskeleton.

There were two genes with levels of expression that differed significantly (*p* = 0) across all treatment groups: LOC662432 (cytochrome P450 9e2, CYP9e2) and LOC107398755 (uncharacterized protein DDB_G0290685-like isoform X1). The expression of LOC662432 was increased and that of LOC107398755 was decreased in all phosphine-resistant adults compared to phosphine-susceptible *T. castaneum* adults regardless of treatment (Figure 1d). LOC107398755 was expressed higher overall in susceptible insects in the PET group at either dose or time exposure.

### 3.2. Differences in Gene Expression from Either Phosphine-Susceptible or -Resistant T. castaneum Adults Among All Treatment Groups

**Figure 2:**
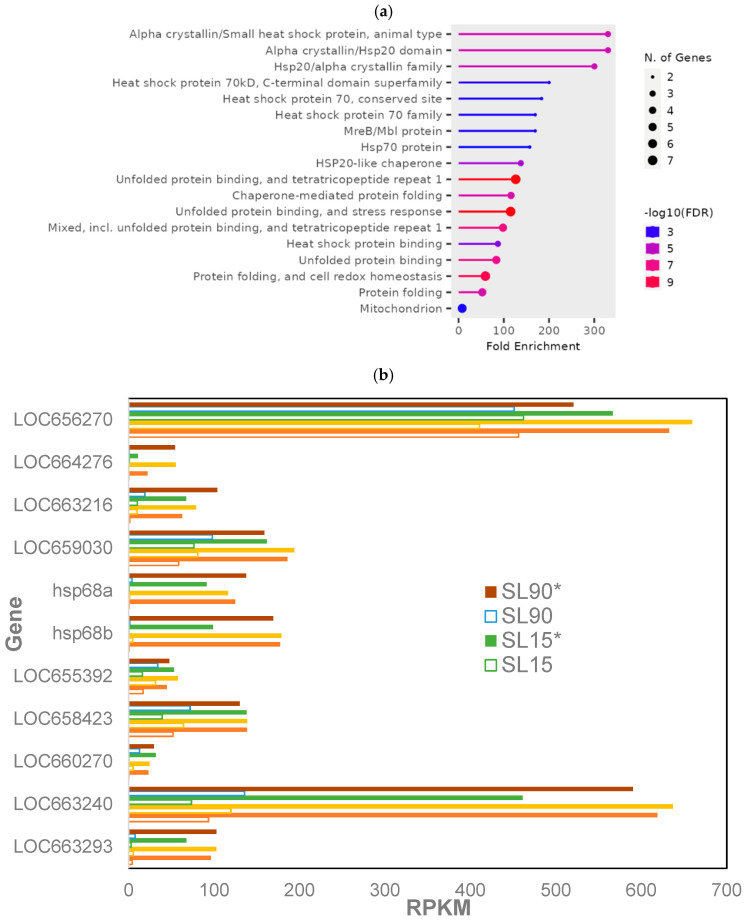

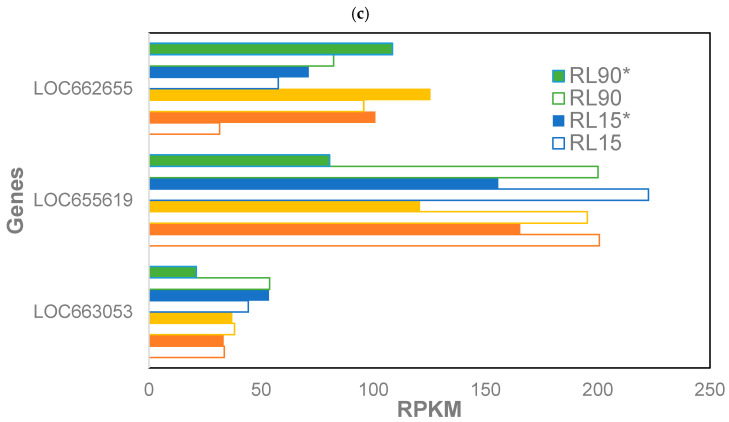
**Differentially expressed genes in phosphine-susceptible *T. castaneum* adults (*p* < 0.05).** (**a**) Enrichment of GO terms in differentially expressed genes in phosphine-susceptible *T. castaneum* adults. (**b**) Expression of HSP in phosphine-susceptible insects, and (**c**) expression of LOC663053, LOC655619 and LOC662655 in phosphine-resistant insects among all treatment groups. Unfilled bars represent gene expression in insects sequenced immediately after phosphine exposure, whereas those sequenced 120 min PET (*) are represented by solid bars.

Overall, 24 genes were expressed differentially among phosphine-susceptible insects in all treatment groups (*p* < 0.05; Supplemental File S2, S_05). The most enriched GO terms were from genes encoding heat shock proteins (HSPs), those related to unfolded protein response (UPR)/tetratricopeptide (TPR)/stress response had the most significant FDR values in phosphine-susceptible insects, whereas the GO term “Mitochondrion” had the greatest number of genes (Figure 2a). HSP genes were expressed at higher levels in the 120 min PET group of susceptible insects regardless of the dose or exposure time (Figure 2b).

Only seven genes had significantly (*p* < 0.05) different expression among phosphine-resistant adults from all treatment groups (Supplemental File S2, R_05). The expressions of selected genes potentially involved in phosphine intoxication were compared (Figure 2c). Two of these genes were LOC663053 (aquaporin-12) and LOC655619 (solute carrier family 25 member 38-A isoform X1). The expression of LOC655619 was decreased in the 120 min PET group of resistant insects, whereas the expression of LOC663053 was increased in this group, but only at the longer exposure time. In contrast, the expression of LOC662655 (peptide methionine sulfoxide reductase) increased in the 120 min PET group of resistant insects regardless of the dose or exposure time.

### 3.3. What Were the Effects of Phosphine Exposure Times on T. castaneum Gene Expression?

#### 3.3.1. Differential Gene Expression of Phosphine-Resistant or -Susceptible *T. castaneum* Adults Exposed to a Low or High Dose of Phosphine for 15 min 

**Figure 3:**
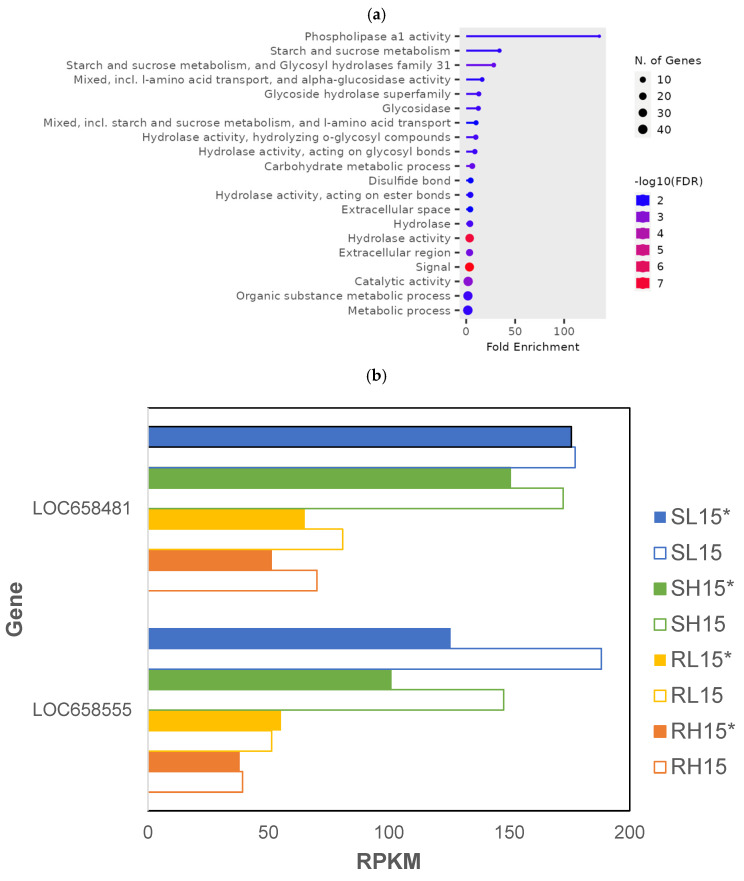
Genes that were differentially expressed in phosphine-resistant and -susceptible *T. castaneum* adults exposed to low or high phosphine dose for 15 min. (**a**) Enriched GO terms from genes differentially expressed in all samples. (**b**) Expression of phospholipase a1 genes LOC658481 and LOC658555; solid bars (*) are insects sequenced after 120 min PET, whereas those immediately sequenced after phosphine exposure are represented by unfilled bars.

There were 147 genes that were differentially expressed in *T. castaneum* adults exposed to phosphine at a low or high dose for 15 min (*p* < 0.01; Supplemental File S2, RS_15_01). The highest fold enrichment of GO terms was found in genes encoding phospholipase a1 (pLA1), whereas GO terms “hydrolase” and “signal” had the most significant FDR, and those encoding metabolic processes contained the most genes (Figure 3a). The expression of two pLA1 genes, LOC658481 and LOC658555, was lower in phosphine-resistant compared to -susceptible insects (Figure 3b). However, the expression of pLA1 genes decreased overall in insects from both strains sequenced 120 min PET compared to those sequenced immediately after exposure (with the exception of RL15*).

#### 3.3.2. Differential Gene Expression of Phosphine-Resistant or -Susceptible *T. castaneum* Adults Exposed to a Low or High Dose of Phosphine for 90 min 

**Figure 4:**
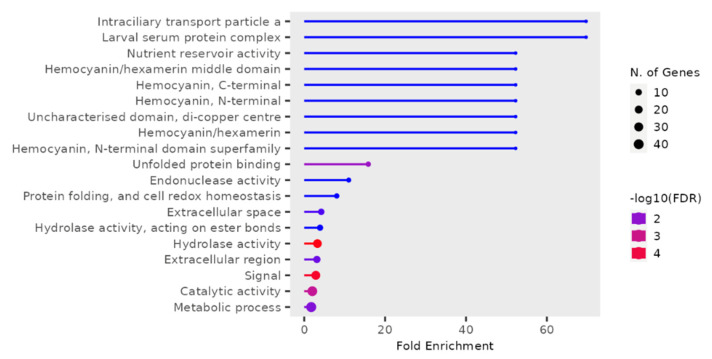
**Differentially expressed genes in phosphine-resistant or -susceptible *T. castaneum* adults exposed to phosphine for 90 min.** Enrichment of GO terms in the 90 min treatment group.

*T. castaneum* adults from either phosphine-resistant or -susceptible strains exposed to a low or high dose of phosphine for 90 min had 190 differentially expressed genes (*p* < 0.01, Supplemental File S2, RS_90_01). The most highly enriched and significant GO terms were mainly from “Intraciliary transport particle a” (related to cytoskeleton transport along microtubules) and “Larval serum protein complex” (storage proteins) (Figure 4); the most genes in this dataset had GO terms “Metabolic activity” and “Catalytic activity”.

#### 3.3.3. Analysis of Reads Aligning to LOC662432 (CYP9e2) 

At longer exposure times, the critical role of LOC662432 (CYP9e2) in the response to phosphine was more apparent, particularly in the phosphine-resistant strain (Figure 1d). After 90 min of exposure to phosphine, the fold increase in LOC662432 expression in phosphine-resistant insects compared to susceptible insects (i.e., RH90/SH90) was 7- to 13-fold higher, and expression was the highest in phosphine-resistant insects selected 120 h PET. Sequence reads from phosphine-susceptible and -resistant *T. castaneum* adults among all treatment groups were aligned to LOC662432 (Supplemental File S3). There were differences in the consensus sequences from phosphine-resistant adults compared to either susceptible sequences or the reference sequence (NC_087397.1). Notably, 15 SNPs were detected in phosphine-resistant reads compared to NC_087397.1 (Table 2).

### 3.4. What Were the Overall Effects of a Low Phosphine Dose on Gene Expression in Phosphine-Susceptible and -Resistant T. castaneum Insects? 

**Figure 5:**
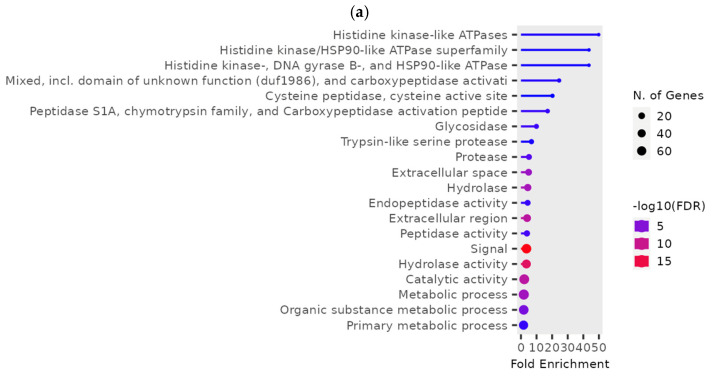

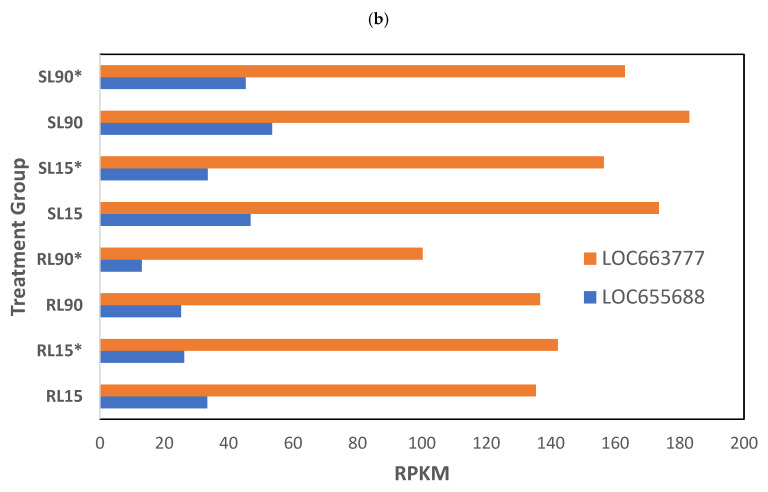
**Differential gene expression in phosphine-susceptible and -resistant *T. castaneum* adults exposed to a low phosphine dose.** (**a**) Enriched GO terms in differentially expressed genes from insects exposed to a low phosphine dose (*p* < 0.01). (**b**) Expression of ATPase subunit genes LOC663777 and LOC655688. Groups with asterisks were sampled 120 min PET.

At a low phosphine dose, the expression of 246 genes was significantly different (*p* < 0.01) in phosphine-susceptible and -resistant *T. castaneum* adults (Supplemental File S2, RS_L_01). In this group, genes with the most highly enriched GO terms were related to histidine/kinase-like ATPases, LOC663777 and LOC655688 (Figure 5a). These genes had lower expression in phosphine-resistant compared to -susceptible *T. castaneum* adults, and this trend was even more apparent in low-dose-exposed insects with longer exposure times (Figure 5b). In agreement with previous analyses, the parent GO terms “signal” and “hydrolases” had the more significant FDR values and higher numbers of genes, reflecting the overall downregulation of gene expression in phosphine-resistant vs. phosphine-susceptible *T. castaneum* adults.

### 3.5. What Were the Overall Effects of a High Phosphine Dose on Gene Expression in Phosphine-Susceptible and -Resistant T. castaneum Insects? 

**Figure 6:**
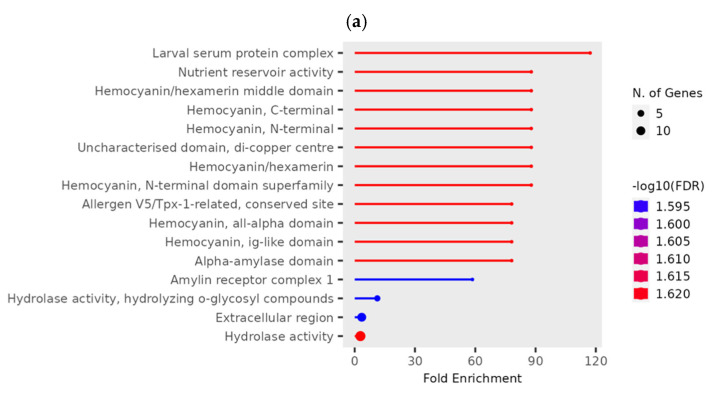

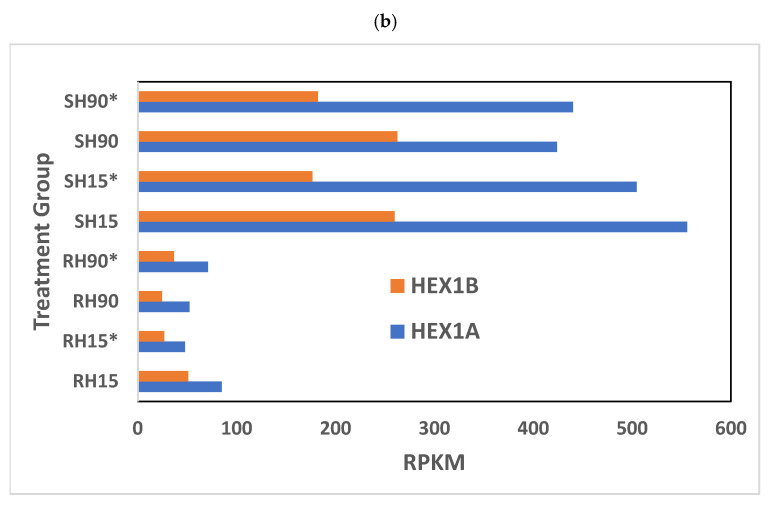
**Differential gene expression of phosphine-susceptible and -resistant *T. castaneum* exposed to a high phosphine dose.** (**a**) Enriched GO terms in genes from insects exposed to a high phosphine dose. (**b**) Hexamerin 1A and 1B gene expression. Groups with asterisks were sampled 120 min PET.

At a high phosphine dose, the expression of 91 genes was significantly different (*p* < 0.01) in phosphine-susceptible and -resistant *T. castaneum* adults (Supplemental File S2, RS_H_01). The GO term “Larval serum protein complex” and those terms related to hemocyanin were more enriched and had more significant FDR values (Figure 6a). As in previous comparisons, the GO term “hydrolase activity” also had significant FDR values and contained the highest number of genes. The expression of the storage proteins hexamerins A and B was lower in phosphine-resistant insects compared to phosphine-susceptible ones (Figure 6b).

### 3.6. Which Genes Were Differentially Expressed After 120 PET in T. castaneum Adults?

**Figure 7:**
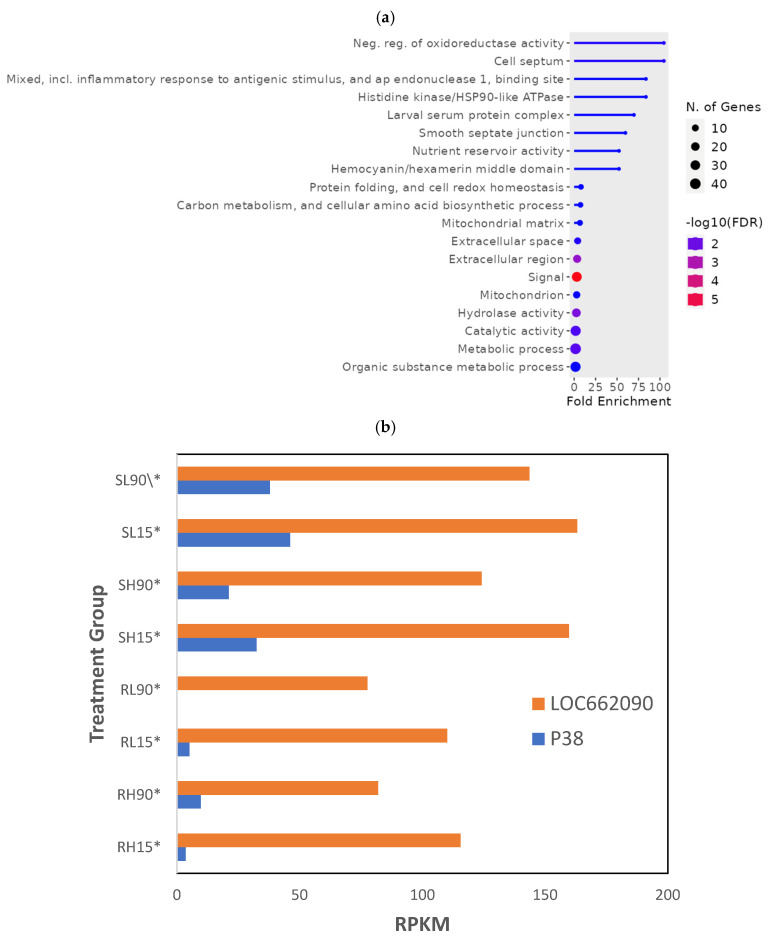
**Genes differentially expressed in *T. castaneum* adults sequenced 120 min PET.** (**a**) Enriched GO termsf in sequences from *T. castaneum* adults collected 120 min PET (*). (**b**) LOC662090 (PDK) and p38 gene expression in phosphine-susceptible and -resistant *T. castaneum* adults.

Overall, 145 genes from all 120 min PET treatment groups were significantly different (*p* < 0.01; Supplemental File S2, RS_PET_01) To fully understand the differences in phosphine-susceptible and -resistant *T. castaneum* adults after that recovery time, we compared genes that decreased (112) and increased (35) overall in phosphine-resistant adults. In the decreased gene expression set, the most enriched GO term was “Neg. reg. of oxidoreductase activity”, the term with the most significant FDR was “signal”, and “Metabolic process” had the most genes (Figure 7a). There was no enrichment of GO terms in the increased gene expression set of phosphine-resistant adults sequenced 120 min PET.

One of the genes from most enriched GO terms was LOC662090 (PDK), annotated as “pyruvate dehydrogenase (acetyl-transferring) kinase, mitochondrial”. The PDK gene encodes a mitochondrial enzyme that regulates glucose oxidation by phosphorylating and inhibiting the pyruvate dehydrogenase (PDH) enzyme, which converts pyruvate into acetyl-CoA for entry into the citric acid cycle. PDK was expressed at lower levels in phosphine-resistant compared to -susceptible *T. castaneum* adults and was further reduced at the higher doses of phosphine in both insect groups (Figure 7b). Another gene in the enrichment group was p38, also known as MAPK14 (mitogen-activated protein kinase 14) that encodes a protein crucial in cellular stress responses. The p38 gene was expressed at lower levels in phosphine-resistant compared to -susceptible insects.

### 3.7. Expression of Phosphine Resistance Genes dld and cyt-b5-r in Phosphine-Susceptible and -Resistant T. castaneum Adults 

**Figure 8:**
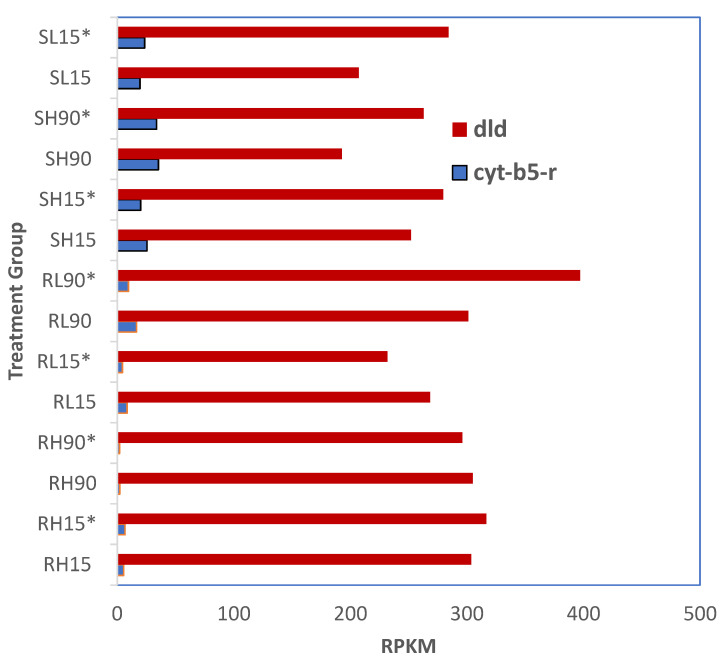
**Differential expression of *cyt-b5-r* and *dld* in phosphine-resistant and -susceptible *T. castaneum* adults.** Groups with asterisks were sampled 120 min PET.

There were no significant differences in the gene expression of *dld* and *cyt-b5-r* in phosphine-susceptible or -resistant *T. castaneum* adults among all treatments (*p* < 0.05, Figure 8). Overall, the expression of *dld* was higher in susceptible adults sequenced 120 min after phosphine exposure at either dose or time; however, this trend was only apparent in resistant adults at the low dose and longer exposure time. The expression of *cyt-b5-r* was higher in susceptible compared to resistant adults.

### 3.8. How Are Differentially Expressed Long Non-Coding RNAs (lncRNAs) Related to Phosphine Sensitivity or Resistance? 

**Figure 9:**
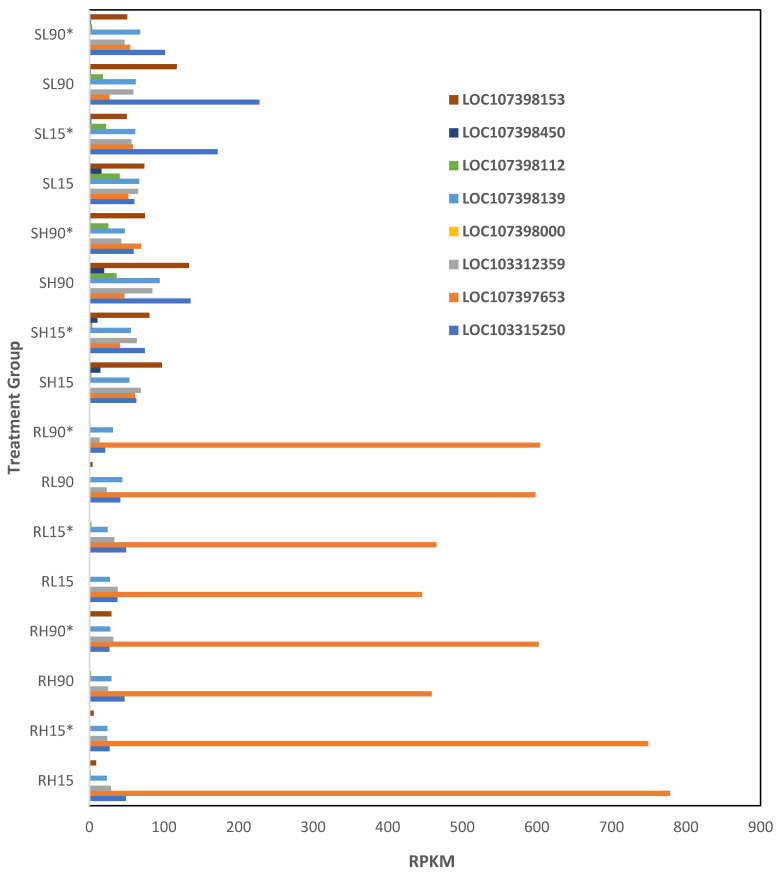
**Differential expression of ncRNAs in phosphine-resistant compared to -susceptible *T. castaneum* adults.** Groups with asterisks were sampled 120 min PET.

Eight lncRNAs were differentially expressed (*p* < 0.01) among phosphine-susceptible and -resistant insects (Supplemental File S2, ncRNA). The expression of the lncRNA LOC107397653 and LOC107398000 was higher in phosphine-resistant compared to phosphine-susceptible *T. castaneum* adults (Figure 9). Alternatively, LOC103315250, LOC103312359, LOC107398139, LOC107398112, and LOC107398153 were decreased in expression in phosphine-resistant compared to -susceptible insects. However, we were unable to identify potential genes that may be affected if these are cis-acting lncRNA.

## 4. Discussion

We evaluated differences in gene expression among *T. castaneum* adults that are genetically distinct and phenotypically different in terms of tolerance to phosphine (i.e., phosphine-susceptible and -resistant) and show different outcomes (i.e., mortality, narcosis) in response to treatments (i.e., low/high phosphine doses, short/long exposure times). In these complex analyses, it can be difficult to determine genes that are mutated and thus responsible for the response versus those that are integrated into metabolic pathways of mutated genes (summarized in Supplemental File S2, summary). For example, in the analysis of all treatment groups with phosphine-susceptible and -resistant *T. castaneum* adults, we detected the differential expression of genes with GO terms “acyl CoA dehydrogenase/fatty acid oxidation” and “histidine kinase/HSP90-like ATPase”. Based on previous studies [8,15], these genes may be affected by mutated genes in the direct response to phosphine dose and/or the time of exposure. We found that histidine/kinase-like ATPases had lower expression in phosphine-resistant compared to -susceptible *T. castaneum* adults exposed to phosphine at the lower dose and thus may be responding to an overall decrease in energy production. The expression of genes encoding hexamerin storage proteins was also decreased in phosphine-resistant *T. castaneum* adults exposed to the higher dose of phosphine, possibly an effort to conserve or redirect energy.

Other GO terms like “a-amylase”, “carbon metabolism” and “hydrolases” may correlate to the decreased respiration that has been identified as a resistance factor for phosphine intoxication in some studies [16,17]. GO terms from genes including glycosidases, lipases, and hydrolases were enriched in differentially expressed gene datasets. These genes are likely responding to the shift in metabolism occurring when insects are exposed to phosphine. Related genes include pLA1, which was downregulated in phosphine-resistant insects and is involved in processes such as fat body metabolism [30]. However, pLA1 expression increased in both phosphine-susceptible and -resistant insects under phosphine exposure, and this may correlate to the higher lipid content observed in phosphine-resistant strains of *T. castaneum* [31]. Since a major toxicity factor coming from phosphine exposure is lipid peroxidation, the increased expression of lipases such as pLA1 could be an adaptive effort to compensate for decreased lipid metabolism.

Genes that potentially contribute to recovery may provide insight into phosphine resistance. These genes include DDB_G0290685-like isoform X1, which encodes an uncharacterized protein that was expressed at higher levels in resistant insects from the PET groups. Despite working with a relatively mature insect genome sequence, many of the significant differentially expressed genes were not annotated and thus lacked functional details. This lack of information also stymied a previous gene expression study of phosphine resistance in *T. castaneum* [8]. However, given the relative importance of DDB_G0290685-like isoform X1 in phosphine recovery, the function of this gene is an active area of research. The expression of another gene important to recovery, LOC663053 (aquaporin-12), was decreased in phosphine-resistant insects that recovered from phosphine exposure at either the dose or exposure time. This gene is found in the hindgut–Malpighian tubule complex and serves as a water channel in the intestinal tract [32]. Calcitonin receptors were also downregulated in the phosphine-resistant strain; these receptors affect salt and water transport in the Malpighian tubules. These data correlate with our previous observations of downregulated anti-diuretic factors (ADFs) in phosphine-resistant *T. castaneum* at a lower dose but longer exposure time (200 ppm, 20 h; [8]). We speculated that phosphine-resistant insects may display increased diuresis, similar to what occurs in controversial treatments for phosphine exposure in mammals [33]. If that is true, increased water secretion may be beneficial to the survival of phosphine exposure.

LOC655619 (solute carrier family 25 member 38-A isoform X1) is found in the inner mitochondrial membrane and is predicted to be involved in glycine import into mitochondria. This gene was also decreased in expression in insects in the phosphine-resistant 120 min PET group, and the lower importation of glycine into the mitochondria may be the result of lower *dld* expression, as *dld* functions as the L-protein of the glycine cleavage system in mitochondria and serves to control the buildup of toxic amounts of glycine. The expression of another gene, LOC662655 (peptide methionine sulfoxide reductase), increased in the phosphine-resistant PET group regardless of dose or exposure time; this gene encodes an enzyme that repairs damage to proteins caused by reactive oxygen and nitrogen intermediates [34].

Enriched GO terms from HSP and UPR genes were found in phosphine-susceptible *T. castaneum* PET adults. The expression of 11 HSPs was increased in susceptible *T. castaneum* PET adults regardless of the dose or time of exposure, suggesting that they were responding to the cellular stress of phosphine intoxication. Similarly, GO terms associated with UPR/TPR were the most significant among genes in phosphine-susceptible *T. castaneum* in the 120 min PET group and included chaperone proteins that maintain the integrity of the endoplasmic reticulum (ER) and prevent oxidative stress [35]. TPR proteins are a family of proteins in the UPR that regulate protein organization and homeostasis within the ER [36].

Phosphine-susceptible *T. castaneum* adults had higher expression of LOC103312224 (FIBCD1) than phosphine-resistant insects, and the expression of this gene was notably elevated at the higher phosphine dose. FIBCD1 is a member of the fibrinogen-related domain (FReD) protein family that recognizes acetylation in chitin, contributing to pattern recognition and immunity [37,38]. The difference in the expression of this gene between phosphine-susceptible and -resistant insects could indicate that it is a possible candidate for a phosphine-resistance marker.

LOC662432, encoding CYP9e2, was more highly expressed in phosphine-resistant *T. castaneum* adults under all treatment conditions, and was expressed at even higher levels in phosphine-resistant adults that survived exposure. The differential expression of a number of cytochrome P450 (CYP) genes also was significant in our previous study of gene expression in these two *T. castaneum* strains [8]. In that study, dose and exposure time were based on the LD_50_ of each strain: for the susceptible insects, the dose was 0.65 ppm for 8 h, and for resistant insects, the dose was 200 ppm for 20 h. Under those conditions, the expression of 44 CYP genes was higher in phosphine-resistant compared to -susceptible adults (14 more than 2-fold higher, and 5 significantly increased at *p* < 0.05 under phosphine exposure); CYP9Ad1 was 8.84-fold higher in the resistant strain compared to the susceptible strain, and CYP6 genes were also highly expressed. Sequencing in the previous study was performed with 454, one of the early next-generation sequencing platforms, whereas sequencing in the present study was performed with Illumina, providing increased accuracy and with higher coverage. The difference in dose/time/sequencing platform and treatment groups in the present study also likely contributed to the clear distinction of CYP9e2 as a major factor in phosphine resistance in *T. castaneum.* Lower phosphine exposure times at higher doses already are the basis for phenotypic phosphine test kits [21,22].

Cytochrome P450 genes are constitutively overexpressed in insecticide-resistant phenotypes, leading to the enhanced metabolic detoxification of insecticides, and remain a key factor underlying insecticide resistance in *T. castaneum* and other insect species [39]. Reports of the increased expression of CYP9e2 in insects exposed to various insecticides include: *Plutella xylostella*, where CYP9e2 transcript levels were elevated 4.7-fold upon exposure to spinosad [40]; *Pectinophora gossypiella,* with a 4.2- to 13.2-fold increase in CYP9E2 expression in Bt-resistant populations [41]; and *Leptinotarsa decemlineata,* where the silencing of CYP9e2 expression led to increased susceptibility to clothianidin and spinosad [42,43], and where the knockdown of upregulated CYP 9e2 and a long non-coding RNA led to increased spinosad mortality [43]. CYP9e2 is implicated in the detoxification of thiacloprid in honeybees [44]. CYP9e2 is not only involved in detoxifying insecticides but also plays a role in broader physiological responses. For example, CYP9e2 is activated in response to immune challenges in honeybees, suggesting a dual role in detoxification and immune responses [44]. The expression of CYP9e2 can be modulated by factors such as air pollution and chemical exposure [44,45]. In fact, another CYP9e2 gene in *T. castaneum* (LOC103314135) was increased in response to hypoxic conditions and was linked to the increased production of *trehalase* genes [46].

Sequences from CYP9e2 in phosphine-resistant *T. castaneum* had 10 SNPs compared to the *T. castaneum* reference sequence for LOC662432, but the exact mechanism of how CYP genes contribute to phosphine resistance is an active area of research in our laboratory. We are investigating whether mutations in CYP genes are common to other phosphine-resistant insects. From a mechanistic standpoint, we are investigating how the mutations contribute to higher expression levels, whether they induce a conformational change in the protein, and if increased expression is due to gene duplication. Regardless, the differences in CYP9e2 sequences can serve as phosphine resistance markers in *T. castaneum*, enabling the development of a quick test for the field evaluation of insect resistance to phosphine.

Data from this study also contribute to insights into the mode of action of phosphine in insects. “Signal” was the most enriched and significant GO term in genes from phosphine-resistant insects from all treatments. This was likely related to the considerable cellular restructuring and altered metabolism occurring in phosphine-exposed insects to avoid phosphine intoxication. Molecular signaling triggers a cellular response, often by binding to a receptor and initiating a signaling pathway. These data are the first report of the significant cellular structural changes that occur in phosphine-resistant insects, as we found enrichment in genes related to cellular restructuring, cytoplasm, and cytoskeleton, especially when insects were exposed to either phosphine dose for 90 min. The smaller subunit of intraciliary transport particle (a) was one of the more enriched GO terms in differentially expressed genes at the 90 min exposure time in both *T. castaneum* strains. This oligomeric protein complex participates in the bidirectional transport of molecules along axonemal microtubules found in insect sperm and in mechano- and chemosensory neurons [47] and may be participating in signaling processes. The most highly enriched GO terms among downregulated genes in phosphine-resistant insects included that encoding “trichohyalin-plectin-homology domain”, which contains trichoplein or mitostatin and is a meiosis-specific nuclear structural protein linked with mitochondrial movement [48]. This protein is associated with the mitochondrial outer membrane, and when downregulated it will enhance mitochondrial movement by binding the mitochondria to actin intermediate filaments [49].

The differential expression of ncRNA in different life stages of *T. castaneum* has been found in RNA-Seq data [50], but little is known about how they affect gene expression in insects. LncRNAs were associated with target mRNA expression in response to terpinen-4-ol fumigation in *T. castaneum*, helping to identify metabolic pathways involved in the mechanism of action of the fumigant [51]. However, the phosphine-induced lncRNAs identified in study did not correlate to any in the terpinen-4-ol fumigation study, and we were unable to locate genes near the chromosomal loci of lncRNAs that may be related to phosphine intoxication. More research is needed on the interactions of ncRNAs and their targets to understand how they may be manipulated to reduce insecticide resistance.

Mutations of *dld* in this phosphine-resistant *T. castaneum* strain were described previously [8], including an additional mutation in the FAD-binding region that may also be related to phosphine resistance and indels that suggest a loss of NADH binding. In that study, we found that phosphine-resistant *T. castaneum* adults had increased expression of *dld*, which was further increased after 20 h of exposure [8]. However, in the present study, no significant increase in *dld* expression was observed at 15 or 90 min of exposure time. The leading hypothesis is that insects that carry *dld* and *cyt-b5-r* mutations have high levels of resistance, and thus differences in gene expression are due to the downstream metabolic effects of these gene disruptions. However, cellular restructuring has not been reported in phosphine intoxication, and thus we cannot comment on whether our observations are a direct result of the mutations. Therefore, an alternate hypothesis is that other mutations may contribute to phosphine resistance, including differences in regulatory elements, such as lncRNAs. Presumably, these lncRNAs are involved in regulating the differential expression of genes in response to *T. castaneum* phosphine exposure, and thus may be a target in decreasing resistance to phosphine intoxication in insects.

The parallel occurrence of the genes identified in this study, which are differentially expressed in phosphine-resistant insects, and previously described phosphine resistance markers may contribute to the creation of a rapid diagnostic tool that is based on compensatory metabolic imbalances after phosphine exposure. Hence, as initially postulated, the two exposure times and phosphine concentrations revealed additional metabolic mechanisms that are directly detectable and can be further evaluated as indicators of resistance. In this study, insects were exposed to phosphine immediately prior to freezing for molecular analyses, and hence these results correlate with exposure and behavioral changes at the specific exposure time, as demonstrated by [5]. The current diagnostic tool, which is based only on the immobilization of insects after exposure to phosphine, is subjective and based on personal observations [23]. From a practical point of view, a field diagnostic kit that analyzes individual insects for molecular markers for phosphine resistance would be useful for fumigators, as it can be performed without the need to generate phosphine. The data from this study provide additional insect genetic markers that can be evaluated in a rapid test for assessing phosphine resistance on site.

## 5. Conclusions

Genetic differences in *T. castaneum* adults, exposed to a low and high dose of phosphine for a short or long period of time, were identified through RNA-Seq. Differences were mostly found in the expression levels of genes related to metabolism, signaling, and cellular structure. While some of these genes can be explored for resistance markers to phosphine, one gene in particular, CYP9e2, appears to be consistently upregulated in phosphine-resistant *T. castaneum* adults and its level further increases in response to phosphine exposure. These data will be useful for developing an in-field rapid diagnostic tool for the detection of phosphine-resistant insects.

## Figures and Tables

**Table 1:** **Abbreviations for treatment groups in this study.** R = phosphine-resistant and S = phosphine-susceptible *T. castaneum* adults; H = high-dose (3000 ppm) and L = low-dose (1000 ppm) phosphine exposure; 15 or 90 min exposure time; collected at 0 or 120 min (*) post-exposure time (PET).

Abbreviation	Strain	Dose (ppm)	Time (min)	PET
SH15	Susceptible	High (3000)	15	0
SH15 *				120
SH90			90	0
SH90 *				120
SL15		Low (1000)	15	0
SL15 *				120
SL90			90	0
SL90 *				120
RH15	Resistant	High (3000)	15	0
RH15 *				120
RH90			90	0
RH90 *				120
RL15		Low (1000)	15	0
RL15 *				120
RL90			90	0
RL90 *				120

**Table 2:** Variants found in the consensus sequence of reads from phosphine-resistant *T. castaneum* adults aligning to LOC662432 in the reference sequence NC_087397.1.

NC_087397.1 Reference Position	Variants from LOC662432 in Phosphine-Resistant Insects
450	T > C
602	C > T
605	G > A
653	C > T
860	C > T
919	A > G
1059	A > C
1498	G > A
1530	G > A
1683	G > A
1746	T > C
1748	T > C
1750	C > G
1788	A > T
1806	A > G
1835	G > T

## Data Availability

All data have been publicly archived as noted in Materials and Methods at NCBI SRA SRR25097208—SRR25097407.

## Supplementary Materials

The following supporting information can be downloaded at: https://www.mdpi.com/article/10.3390/genes16030324/s1, Supplemental File S1, Read statistics from all samples used in this study; Supplemental File S2, Statistically significant gene groups discussed in this study; Supplemental File S3, Alignment of CYP9e2 genes from susceptible and resistant insects compared to reference sequence NC_087397.1.

## Abbreviations

The following abbreviations are used in this manuscript:

CYP9e2	cytochrome P450 9e2
cyt-b5-r	cytochrome b5 reductase
FIBCD1	fibrinogen C domain-containing protein 1
LD50 lncRNA	the dose leading to 50% mortality long non-coding RNA
PDH	pyruvate dehydrogenase
PET PDK	post-exposure time (phosphine) pyruvate dehydrogenase (acetyl-transferring) kinase
PTT	Phosphine Tolerance Test
rph1	genetic locus in insects responsible for weak resistance to phosphine
rph2	genetic locus in insects responsible for strong resistance to phosphine
TPR	tetratricopeptide
UPR	unfolded protein response
