# Supplementary material for: Short Exposures to Phosphine Trigger Differential Gene Expression in Phosphine-Susceptible and -Resistant Strains of Tribolium castaneum
Source: Genes (Basel). 2025 Mar 10;16(3):324. doi: 10.3390/genes16030324 (PMC11942322; doi:10.3390/genes16030324)
Supplement: Supplementary file 1 [file genes-16-00324-s001.zip › Supplemental File S3.pdf]

```
CLUSTAL 2.1 Multiple Sequence Alignments


Sequence type explicitly set to Protein
Sequence format is Pearson
Sequence 1: LOC662432_susceptible_consensus  1943 aa
Sequence 2: LOC662432_resistant_consensus    1943 aa
Sequence 3: NC_087397.1_1583671-1585481      1811 aa
Start of Pairwise alignments
Aligning...



(Partial alignment)
Sequences (1:2) Aligned. Score: 54.7092
(Partial alignment)
Sequences (1:3) Aligned. Score: 60.7951
(Partial alignment)
Sequences (2:3) Aligned. Score: 60.6847
Guide tree file created:   [clustalw.dnd]

There are 2 groups
Start of Multiple Alignment

Aligning...
Group 1: Sequences:   2      Score:34760
Group 2: Sequences:   3      Score:21963
Alignment Score 37030

CLUSTAL-Alignment file created  [clustalw.aln]
```

---

clustalw.aln

```
CLUSTAL 2.1 multiple sequence alignment
```

R = A/G
Y = C/T

```
LOC662432_susceptible_consensu      AAATGCTCCTTAAGAATATCTTACATAATCAAATRATCAGTTTAATGGAA
NC_087397.1_1583671-1585481         ---------TTAAGAATATCTTACATAATCAAATAATCAGTTTAATGGAA
LOC662432_resistant_consensus       AAATGCTCCTTAAGAATATCTTACATAATCAAATAATCAGTTTAATGGAA
                                             ************************* ***************

LOC662432_susceptible_consensu      GACTTGAGAGAAACATGTTGCTTTTACTATTAGGCACACTAATCGTGATT
NC_087397.1_1583671-1585481         GACTTGAGAGAAACATGTTGCTTTTACTATTAGGCACACTAATCGTGATT
LOC662432_resistant_consensus       GACTTGAGAGAAACATGTTGCTTTTACTATTAGGCACACTAATCGTGATT
                                    **************************************************

LOC662432_susceptible_consensu      YTAGTTTTATACCTCGTTAATCTATCGACAAAGAATTTTGATCATTGGGA
NC_087397.1_1583671-1585481         CTAGTTTTATACCTCGTTAATCTATCGACAAAGAATTTTGATCATTGGGA
LOC662432_resistant_consensus       YTAGTTTTATACCTCGTTAATCTATCGACAAAGAATTTTGATCATTGGGA
                                     *************************************************

LOC662432_susceptible_consensu      AAAACGCAGAGTGAAATATGTGAAACCGTTCCCATTTGTTGGAAACTTAT
NC_087397.1_1583671-1585481         AAAACGCAGAGTGAAATATGTGAAACCGTTCCCATTTGTTGGAAACTTAT
LOC662432_resistant_consensus       AAAACGCAGAGTGAAATATGTGAAACCGTTCCCATTTGTTGGAAACTTAT
                                    **************************************************

LOC662432_susceptible_consensu      TACCAACGGTGTTGAAAACAAAATCAACCGCTGAACTTATCCAACACCTA
NC_087397.1_1583671-1585481         TACCAACGGTGTTGAAAACAAAATCAACCGCTGAACTTATCCAACACCTA
```

```
LOC662432_resistant_consensus       TACCAACGGTGTTGAAAACAAAATCAACCGCTGAACTTATCCAACACCTA
                                    **************************************************

LOC662432_susceptible_consensu      TACAAGGCTTTTCCTAATGAAAGGTTCGTGGGAATTTTTCAATTTAATAC
NC_087397.1_1583671-1585481         TACAAGGCTTTTCCTAATGAAAGGTTCGTGGGAATTTTTCAATTTAATAC
LOC662432_resistant_consensus       TACAAGGCTTTTCCTAATGAAAGGTTCGTGGGAATTTTTCAATTTAATAC
                                    **************************************************

LOC662432_susceptible_consensu      TCCCATTTTGTTGATACGAGACCCTGAATTAATCAAGTCTATTGCGATAA
NC_087397.1_1583671-1585481         TCCCATTTTGTTGATACGAGACCCTGAATTAATCAAGTCTATTGCGATAA
LOC662432_resistant_consensus       TCCCATTTTGTTGATACGAGACCCTGAATTAATCAAGTCTATTGCGATAA
                                    **************************************************

LOC662432_susceptible_consensu      AAAACTTCGACAATTTYGTKGACCACTATGGGTTTGCTAATGATGACATT
NC_087397.1_1583671-1585481         AAAACTTCGACAATTTCGTTGACCACTATGGGTTTGCTAATGATGACATT
LOC662432_resistant_consensus       AAAACTTCGACAATTTCGTTGACCACTATGGGTTTGCTAATGATGACATT
                                    **************** **.******************************

LOC662432_susceptible_consensu      GATCCATTATGGGCCAAAAATTTGTTTGCAAGCCAAGGTAAAATTTCTGT
NC_087397.1_1583671-1585481         GATCCATTATGGGCCAAAAATTTGTTTGCAAGCCAAGGTAAAATTTCTGT
LOC662432_resistant_consensus       GATCCATTATGGGCCAAAAATTTGTTTGCAAGCCAAGGTAAAATTTCTGC
                                    ************************************************* 

LOC662432_susceptible_consensu      CTTGTTTATTTTTATTATCATTGGTATTAATAGGGGAACGGTGGCGGGAC
NC_087397.1_1583671-1585481         CTTGTTTATTTTTATTATCATTGGTATTAATAGGGGAACGGTGGCGGGAC
LOC662432_resistant_consensus       CTTGTTTATTTTTATTATCATTGGTATTAATAGGGGAACGGTGGCGGGAC
                                    **************************************************

LOC662432_susceptible_consensu      CTACGTCAAACTTTAAGTCCCGTGTTTACCAGCAGCAAAATGCGAACAAT
NC_087397.1_1583671-1585481         CTACGTCAAACTTTAAGTCCCGTGTTTACCAGCAGCAAAATGCGAACAAT
LOC662432_resistant_consensus       CTACGTCAAACTTTAAGTCCCGTGTTTACCAGCAGCAAAATGCGAACAAT
                                    **************************************************

LOC662432_susceptible_consensu      GTTCGTCCTGATGGACGAATGCGTAAAACAGTTAACTGAATATTTCAAGG
NC_087397.1_1583671-1585481         GTTCGTCCTGATGGACGAATGCGTAAAACAGTTAACTGAATATTTCAAGG
LOC662432_resistant_consensus       GTTCGTCCTGATGGACGAATGCGTAAAACAGTTAACTGAATATTTCAAGG
                                    **************************************************

LOC662432_susceptible_consensu      ACCAGAAGCAGGATGTAATAGACATTGAACTAAAAGACATTTTTTCGAGA
NC_087397.1_1583671-1585481         ACCAGAAGCAGGATGTAATAGACATTGAACTAAAAGACATTTTTTCGAGA
LOC662432_resistant_consensus       ATCAAAAGCAGGATGTAATAGACATTGAACTAAAAGACATTTTTTCGAGA
                                    * **.*********************************************

LOC662432_susceptible_consensu      TACACCACCGATGTGATTGCGACCACAGCCTTCGGAATCAAGGTCGATTC
NC_087397.1_1583671-1585481         TACACCACCGATGTGATTGCGACCACAGCCTTCGGAATCAAGGTCGATTC
LOC662432_resistant_consensus       TATACCACCGATGTGATTGCGACCACAGCCTTCGGAATCAAGGTCGATTC
                                    ** ***********************************************

LOC662432_susceptible_consensu      CTTGAGAAATAGAAATAACGACTTTGTGGTATCAGGACGAGAATTCACCG
NC_087397.1_1583671-1585481         CTTGAGAAATAGAAATAACGACTTTGTGGTATCAGGACGAGAATTCACCG
LOC662432_resistant_consensus       CTTGAGAAATAGAAATAACGACTTTGTGGTATCAGGACGAGAATTCACCG
                                    **************************************************

LOC662432_susceptible_consensu      ATTTTTCAGGATTAAGAGGCCTCGCATTTTTCATTAATGGAAGTTATCCA
NC_087397.1_1583671-1585481         ATTTTTCAGGATTAAGAGGCCTCGCATTTTTCATTAATGGAAGTTATCCA
LOC662432_resistant_consensus       ATTTTTCAGGATTAAGAGGCCTCGCATTTTTCATTAATGGAAGTTATCCA
                                    **************************************************

LOC662432_susceptible_consensu      AGACTTGCCAAAGTAAGCAAACACTTGTAGAATATTTTTCTAATTGWAAT
NC_087397.1_1583671-1585481         AGACTTGCCAAAGTAAGCAAACACTTGTAGAATATTTTTCTAATTGTAAT
LOC662432_resistant_consensus       AGACTTGCCAAAGTAAGCAAACACTTGTAGAATATTTTTCTAATTGTAAT
                                    ********************************************** ***

LOC662432_susceptible_consensu      TTTTAGTTTCTTAATATAAAAATAGTAAGTGATCGCTTGGGTAATTTCTT
NC_087397.1_1583671-1585481         TTTTAGTTTCTTAATATAAAAATAGTAAGTGATCGCTTGGGTAATTTCTT
LOC662432_resistant_consensus       TTTTAGTTTYTTAATATAAAAATAGTAAGTGATCGCTTGGGTAATTTCTT
                                    ********* ****************************************


LOC662432_susceptible_consensu      TCGAACAATAATCAAAGAAACTCTYACTTTACGAGAAGAGAAAAAAATTG
NC_087397.1_1583671-1585481         TCGAACAATAATCAAAGAAACTCTCACTTTACGAGAAGAGAAAAAAATTG
```

```
LOC662432_resistant_consensus      TCGAACAATAATCAAAGAGACTCTCACTTTACGAGAAGAGAAAAAAATTG
                                   ******************.***** *************************

LOC662432_susceptible_consensu     TTCGGCCTGACCTCATCCACTCCCTAATGCTGGCAAGAAAAGGCAAATTA
NC_087397.1_1583671-1585481        TTCGGCCTGACCTCATCCACTCCCTAATGCTGGCAAGAAAAGGCAAATTA
LOC662432_resistant_consensus      TTCGGCCTGACCTCATCCACTCCCTAATGCTGGCAAGAAAAGGCAAATTA
                                   **************************************************

LOC662432_susceptible_consensu     AAATACGAGGAACTAATGGAGTTACCAGAAGCCGGTTTTGCCGCAGTGGA
NC_087397.1_1583671-1585481        AAATACGAGGAACTAATGGAGTTACCAGAAGCCGGTTTTGCCGCAGTGGA
LOC662432_resistant_consensus      AAATACGAGGAACTAATGGAGTTACCAGAAGCCGGTTTTGCCGCAGTGGA
                                   **************************************************

LOC662432_susceptible_consensu     AGAATCCAACTTGACAAAATCTAACAACAACGTAAATTTTTTRACCGATG
NC_087397.1_1583671-1585481        AGAATCCAACTTGACAAAATCTAACAACAACGTAAATTTTTTAACCGATG
LOC662432_resistant_consensus      AGAATCCACCTTGACAAAATCTAACAACAACGTAAATTTTTTAACCGATG
                                   ********.********************************* *******

LOC662432_susceptible_consensu     AGGACATAACAGCCCAAGCTTTGGTCTTCTTCCTTGGCGGTTTCGACACA
NC_087397.1_1583671-1585481        AGGACATAACAGCCCAAGCTTTGGTCTTCTTCCTTGGCGGTTTCGACACA
LOC662432_resistant_consensus      AGGACATAACAGCCCAAGCTTTGGTCTTCTTCCTTGGCGGTTTCGACACA
                                   **************************************************

LOC662432_susceptible_consensu     ACGTCCTCACTGATGTGCTTCGCTGGATACGAACTCGCAATTAACCCCCA
NC_087397.1_1583671-1585481        ACGTCCTCACTGATGTGCTTCGCTGGATACGAACTCGCAATTAACCCCCA
LOC662432_resistant_consensus      ACGTCCTCACTGATGTGCTTCGCTGGATACGAACTCGCAATTAACCCCCA
                                   **************************************************

LOC662432_susceptible_consensu     CATCCAAAAAAGACTCAAAGACGAAGTCCTAGCCACCGACCGCGAATGCA
NC_087397.1_1583671-1585481        CATCCAAAAAAGACTCAAAGACGAAGTCCTAGCCACCGACCGCGAATGCA
LOC662432_resistant_consensus      CATCCAAAAAAGACTCAAAGACGAAGTCCTAGCCACCGACCGCGAATGCA
                                   **************************************************

LOC662432_susceptible_consensu     ACGGCCAAATAACGTACGAAAAACTCCTAAATATGAAATACCTGGACATG
NC_087397.1_1583671-1585481        ACGGCCAAATAACGTACGAAAAACTCCTAAATATGAAATACCTGGACATG
LOC662432_resistant_consensus      ACGGCCAAATAACGTACGAAAAACTCCTAAATATGAAATACCTGGACATG
                                   **************************************************

LOC662432_susceptible_consensu     GTCGTGTCCGAAACGTTACGTAAATGGAACCAAGCCGTTTGGCTCGACCG
NC_087397.1_1583671-1585481        GTCGTGTCCGAAACGTTACGTAAATGGAACCAAGCCGTTTGGCTCGACCG
LOC662432_resistant_consensus      GTCGTGTCCGAAACGTTACGTAAATGGAACCAAGCCGTTTGGCTCGACCG
                                   **************************************************

LOC662432_susceptible_consensu     AAAATGCACCAAAGAAATTGAAATCGAATCGGAAACAAGTGGAGAACCTT
NC_087397.1_1583671-1585481        AAAATGCACCAAAGAAATTGAAATCGAATCGGAAACAAGTGGAGAACCTT
LOC662432_resistant_consensus      AAAATGCACCAAAGAAATTGAAATCGAATCGGAAACAAGTGGAGAACCTT
                                   **************************************************

LOC662432_susceptible_consensu     CAGTTACTCTCAAAGTTGGAGATATCATCTGGATGCCCGCTTATGCCATC
NC_087397.1_1583671-1585481        CAGTTACTCTCAAAGTTGGAGATATCATCTGGATGCCCGCTTATGCCATC
LOC662432_resistant_consensus      CAGTTACTCTCAAAGTTGGAGATATCATCTGGATGCCCGCTTATGCCATC
                                   **************************************************

LOC662432_susceptible_consensu     CATCACGATCCWAAATATTACCCTAACCCGGAACTTTTCGATCCAGARCG
NC_087397.1_1583671-1585481        CATCACGATCCTAAATATTACCCTAACCCGGAACTTTTCGATCCAGAGCG
LOC662432_resistant_consensus      CATCACGATCCTAAATATTACCCTAACCCGGAACTTTTCGATCCAGAACG
                                   *********** *********************************** **

LOC662432_susceptible_consensu     GTTTAGTGACGAAAATAAGGACAAAATTAGAACCGGCACTTATCTACCGT
NC_087397.1_1583671-1585481        GTTTAGTGACGAAAATAAGGACAAAATTAGAACCGGCACTTATCTACCGT
LOC662432_resistant_consensus      GTTTAGTGACGAAAATAAGGACAAAATTAAAACCGGCACTTATCTACCGT
                                   *****************************.********************

LOC662432_susceptible_consensu     TCGGAGTTGGGCCCAGAAATTGTATTGGRTCAAGRTTTGCTTTACTGGAA
NC_087397.1_1583671-1585481        TCGGAGTTGGGCCCAGAAATTGTATTGGGTCAAGATTTGCTTTACTGGAA
LOC662432_resistant_consensus      TCGGAGTTGGGCCCAGAAATTGTATTGGGTCAAGATTTGCTTTACTGGAA
                                   **************************** ***** ***************

LOC662432_susceptible_consensu     ACTAAGCTCTTGTTGTACAACCTTTTGCTCAATTTTGACCTTGTCACCAA
NC_087397.1_1583671-1585481        ACTAAGCTCTTGTTGTACAACCTTTTGCTCAATTTTGACCTTGTCACCAA
```

```
LOC662432_resistant_consensus       ACTAAGCTCTTGTTGTACAACCTTTTGCTCAATTTTGACCTTGTCACCAA
                                    **************************************************


LOC662432_susceptible_consensu      CCACAAAACTGAAAATCCGATTAAGTTACGGARAGACACGCCGTTATTGA
NC_087397.1_1583671-1585481         CCACAAAACTGAAAATCCGATTAAGTTACGGAGAGACACGCCGTTATTGA
LOC662432_resistant_consensus       CCACAAAACTGAAAATCCGATTAAGTTACGGAAAGACACGCCGTTWTTGA
                                    ******************************** ************ ****

LOC662432_susceptible_consensu      TGCCCCAAAATGGGTTTCAAGTGAGTCTTCGAAAAAATGACGCTTTTTCC
NC_087397.1_1583671-1585481         TGCCCCAAAATGGGTTTCAAGTGAGTCTTCGAAAAAATGACGCTTTTTCC
LOC662432_resistant_consensus       TGCCCCAAAATGGGTTTCAAGTGAGTCTTCGAAAAAATGACGCTTCTCCG
                                    ********************************************* * * 

LOC662432_susceptible_consensu      GAAAAATAATCATTTAATTGTTTGTACATATGACCTGAATTATTTAAATA
NC_087397.1_1583671-1585481         GAAAAATAATCATTTAATTGTTTGTACATATGACCTGAATTATTTAAATA
LOC662432_resistant_consensus       GAAAAATAATCATTTAATTGTTTGTACATATGACCTGTATTATTTAAATA
                                    *************************************:************

LOC662432_susceptible_consensu      AATACATCTGTTTGACAGTTTGTTGCTAAATTTCKTCAAATATATTGCTT
NC_087397.1_1583671-1585481         AATACATCTGTTTGACAGTT------------------------------
LOC662432_resistant_consensus       AATACGTCTGTTTGACAGTTTGTTGCTAAATTTCTTCAAATATATTGCTT
                                    *****.**************

LOC662432_susceptible_consensu      TTATCACGTTTATCTTCGAATAAGTGATAATGTRGTTGTAAACACAGTTA
NC_087397.1_1583671-1585481         --------------------------------------------------
LOC662432_resistant_consensus       TTATCACGTTTATCTTCGAATAAGTGATAATGTAGTTGTAAACACAGTTA


LOC662432_susceptible_consensu      TCTAATAGCTGCACATTTGGCAATAAAAATATTCTTAAGCGAA
NC_087397.1_1583671-1585481         -------------------------------------------
LOC662432_resistant_consensus       TCTAATAGCTGCACATTTGGCAATAAAAATATTCTTAAGCGAA
```
